# The Impact of Using Carbohydrate Counting on Managing Diabetic Patients: A Review

**DOI:** 10.7759/cureus.48998

**Published:** 2023-11-18

**Authors:** Sara M H. Ibrahim, Elham A Shahat, Lamar A Amer, Abdullah K Aljohani

**Affiliations:** 1 Physiology, Taibah University, Medina, SAU; 2 Medicine and Surgery, Taibah University, Medina, SAU

**Keywords:** glycaemic index, basic carbohydrate counting, advanced carbohydrate counting, insulin calculations, glycated hemoglobin, carbohydrate counting, diabetes mellitus

## Abstract

Carbohydrate counting (CC) is a meal planning practice for diabetic patients, focusing on tracking the amount of carbohydrates in grams consumed at meals to manage blood glucose (BG) levels. The purpose of this narrative review is to evaluate the impact of CC in helping people with diabetes manage their condition. It reveals that CC offers superior glycemic control and flexibility compared to other food planning techniques. Specifically, when applied to children and teenage patients diagnosed with type 1 diabetes mellitus (T1DM), CC demonstrates the potential for substantial improvements in metabolic control without any adverse effects on weight or increased insulin requirements. In the context of T1DM, the combination of CC and the use of automated bolus calculators (ABCs) contributes to lowering glycated hemoglobin (HbA1c) levels. Furthermore, the study highlights that CC also holds promise in the management of type 2 diabetes mellitus (T2DM). In T2DM patients, adhering to a low glycemic index (GI) diet has proven to be more effective in controlling HbA1c and fasting BG levels compared to a higher GI diet or standard dietary control. This research underscores the evolving significance of CC as a pivotal component in diabetes management, attributed to increased awareness and education among patients. CC emerges as a versatile tool that can benefit individuals with various forms of diabetes by enhancing their glycemic control and overall quality of life. The findings affirm the impact of CC in improving patient outcomes, solidifying its status as a vital strategy in the multifaceted landscape of diabetes care.

## Introduction and background

Diabetes mellitus (DM) is a metabolic disorder characterized by elevated levels of blood glucose (BG) due to compromised insulin secretion, impaired insulin action, or a combination of both [1]. Based on the presence or absence of insulin, diabetes mellitus can be classified into two primary groups: type 1 diabetes mellitus (T1DM) and type 2 diabetes mellitus (T2DM) [2]. T1DM manifests when the pancreatic β cells are damaged by an autoimmune response, leading to a complete deficiency of insulin. It accounts for about 10% of all diabetes cases [2]. The initial manifestations in T1DM patients can vary considerably, including symptoms such as polydipsia, polyuria, enuresis, fatigue, acute exhaustion, polyphagia, sudden weight loss, delayed wound healing, recurrent infections, impaired vision, and severe dehydration and diabetic ketoacidosis. Notably, children often experience more severe symptoms compared to adults [3]. Consequently, individuals diagnosed with T1DM become dependent on external insulin administration [1].

T2DM is a chronic condition characterized by decreased insulin sensitivity, known as "insulin resistance," in the liver, muscles, and adipose tissue, accompanied by reduced pancreatic cell secretory activity. Even in the most severe manifestations of T2DM, the pancreas retains the ability to generate insulin; however, the quantity produced is inadequate for sustaining BG levels within the established normal range. The initial stages of T2DM may exhibit minimal symptoms, leading to underdiagnosis for prolonged periods, particularly in regions where routine checkups without symptoms are not part of the norm. Prolonged hyperglycemia and insulin resistance in T2DM during this undiagnosed phase may expose these individuals to an elevated risk of long-term complications such as obesity, nephropathy, essential hypertension, ovarian hyperandrogenism, premature adrenarche, nonalcoholic fatty liver disease, dyslipidemia, and systemic inflammation [2,3].

Carbohydrate counting (CC) was first introduced as a meal-planning technique for people with T1DM. The correct bolus insulin dosage is determined by considering both the total carbohydrate intake per meal and the insulin-to-carbohydrate ratio (ICR). These two factors are utilized to calculate the precise amount of insulin required for the effective management of BG levels after each meal. Evidence suggests that CC improves metabolic control and lowers glycosylated hemoglobin levels (HbA1c) [4]. Improving glycemic control in T1DM patients may delay T1DM-related long-term microvascular problems [5]. A high glycemic index (GI) food comprises carbohydrates that break down fast during digestion and are quickly absorbed into the bloodstream. On the other hand, a low-GI index diet, which comprises carbohydrates that take a longer time to be digested and absorbed, may help glycemic control by improving insulin sensitivity, minimizing BG variations, and lowering daily insulin needs [6,7]. The prevalence of DM is remarkably high, making it one of the most common chronic conditions found in both developed and developing countries. In Saudi Arabia, it has the second-highest prevalence among adults [8]. The aim of this narrative review was to evaluate the impact of using CC in managing diabetic patients.

## Review

DM is a complicated, long-term illness that necessitates continuous medical attention as well as multifaceted risk-reduction methods that go beyond glucose management. There is substantial evidence to support a variety of therapies to improve diabetes outcomes [9]. Diabetes has a global impact, affecting approximately 422 million individuals worldwide, with a notable concentration in low- and middle-income countries. It directly contributes to 1.6 million deaths annually. In recent decades, there has been a substantial increase in both the number of diabetes cases and their prevalence [10,11]. Diabetes currently stands as the fifth leading cause of death worldwide. Anticipated data indicate that this number will elevate to 552 million by the year 2030 [12,13]. Diabetes commonly leads to complications, including diabetic retinopathy, nephropathy, neuropathy, heart attacks, strokes, and lower limb amputation. Notably, there was a 5% increase in diabetes-related premature mortality between 2000 and 2016. Diabetes is expected to have caused 1.5 million deaths in 2019. In 2012, elevated BG levels were accountable for a further 2.2 million mortalities [8,12,14]. The World Health Organization (WHO) reports that Saudi Arabia has the second-highest prevalence rate of diabetes in the Middle East and the seventh-highest globally. There are around 7 million diabetic patients in Saudi Arabia [12,15].

Carbohydrate counting

T1DM and T2DM are chronic illnesses that affect daily activities and involve long-term lifestyle modifications. Therefore, the importance of education in obtaining and maintaining a healthy lifestyle and treating diabetes should not be underestimated. Early educational intervention at the time of diagnosis, as well as continuous training, is required [16]. Educating patients on how to modify prandial insulin dosing based on carbohydrate intake, preprandial glucose levels, and anticipated exercise can be highly beneficial and is advised for the majority of patients [17]. 

The most significant factor affecting postprandial BG levels is carbohydrate consumption. Precise CC is crucial for determining the accurate insulin dose required to regulate postprandial glycemic levels effectively [4].

CC is a meal-planning technique designed specifically for individuals with diabetes, centering on the management of carbohydrate intake. Over the last decade, the introduction of insulin analogues and insulin pump therapy has led to increased popularity in CC. The use of CC in meal planning for children is important to improve metabolic management, growth, and development. Many physicians have found CC to be an effective educational tool for the management of children with diabetes [18]. Bawazeer et al. presented encouraging findings from their investigation into the competency of CC knowledge among adults with T1DM in KSA [19]. Medical nutrition therapy guidelines suggest that individuals dealing with T1DM are advised to acquire knowledge of CC or other methods based on practical experience to enhance the management of BG levels [1]. CC is categorized internationally into two levels, each having distinct learning objectives and gradually becoming more complex: basic CC (BCC) and advanced CC (ACC) [20].

The BCC approach is a strategic method designed to enhance individuals' comprehension of carbohydrate consumption for improved diabetes management. This educational process teaches individuals to regulate carbohydrate intake, involving identifying carb-rich foods, interpreting nutritional labels, and accurately estimating portions. It emphasizes consistency in the consumption of carbohydrate-containing foods, considering timing, type, amount, and distribution to maintain stable BG levels. The method is based on the principle that 15 grams of carbs equal one portion, contributing to overall glycemic control and steady BG levels. In comparison, the ACC approach is targeted at individuals proficient in BCC, engaging in intensive insulin therapy, and motivated to acquire skills for adjusting insulin dosages according to carbohydrate intake. ACC entails employing insulin dose calculations using the insulin-to-carbohydrate ratio (ICR) and insulin sensitivity factor (ISF). This approach involves various formulas or techniques to determine insulin dosage needs based on ICR and ISF, with variations depending on specific conditions under which CC is applied [20,21].

A carbohydrate that is combined with protein and fat may have a lower GI than a carbohydrate consumed alone [22]. Lifestyle changes, such as dietary interventions and pharmaceutical therapies, are frequently used in diabetes management strategies. There is also evidence that long-term intake of high GI and glycemic load (GL) foods has consequences for metabolism and health, such as chronic hyperglycemia and hyperinsulinemia, which can lead to insulin resistance and diabetes [6]. On the low GI diet, the incidence of moderate hypoglycemia was considerably greater than on the high GI diet, suggesting that insulin doses may need to be decreased even more with low GI meals [23]. The American Diabetes Association (ADA) suggests that individuals with diabetes who have acquired expertise in CC can also be educated on the glycemic effects of protein and fat. However, the patients should have a thorough understanding of insulin calculations, GI, and GL [7].

GI and GL

The relative role of the amount and type of carbohydrate in regulating the BG response to meals is a significant subject of conflict. The term "carbohydrate type" has become interchangeable with the term "glycemic index," which is a way of defining a food's glycemic effect [23].

Foods having a high GI value are digested and absorbed more quickly, resulting in more variations in BG per unit of carbohydrate than foods with a lower GI value [24]. Different foods and their impact on BG and insulin levels from various diets are variable, suggesting that different carbohydrate sources induce variable glycemic responses: GL and GI [25]. In the postprandial phase, consuming a high-GI food containing the same amount of carbohydrates as a low-GI food leads to a greater area under the glucose curve [26].

Carneiro et al.'s research illustrates the impact of variable GI on BG levels [27]. The ingestion of high-GI food can lead to lower BG concentrations during the late postprandial phase (two to three hours after a meal), as compared to the consumption of low-GI food, due to the stimulated insulin response [28]. Decreased insulin demand, enhanced BG control, and lower blood lipid concentrations are among the potential health advantages associated with reducing dietary GI [24].

Populations in China and the United States reported that women who consumed a high-GI diet were more likely to develop T2DM than women who ate a low-GI diet [6]. High-GI meals may raise the risk of T2DM through two routes, according to metabolic data. A high-GI diet causes a relatively high BG concentration and insulin requirement. Over time, this increased insulin demand can lead to a significant decline in pancreatic function [25]. It can also cause insulin resistance directly by increasing postprandial fatty acid synthesis [28]. The amount of carbohydrates and the GI of the food are considered while calculating GL. The GL of a food can be calculated by multiplying the GI value by the grams of carbohydrates, then dividing by 100. Individuals with diabetes should also be knowledgeable about the GI [22,29].

Insulin calculation

The ICR is different for each person and is determined by their insulin sensitivity, or how many grams of carbohydrates 1 unit of insulin covers. ICR calculates insulin requirements at mealtimes based on the carbs that will be consumed, their BG level, and their expected physical activity [4].

Calculation of the appropriate insulin requirement involves using BG measurements before and after meals, as well as during the night. Individual determinations were made based on goal BG, ICR, and ISF. The ICR was calculated by dividing the total daily dose (TDD) of insulin by a coefficient of 500, while the ISF was calculated by dividing the TDD of insulin by a coefficient of 1800 [1].

The impact of time on HbA1c changes is a crucial consideration that should not be neglected. ICR fluctuates more in children than in adults, which is attributed to the child's daily activity changing. Another factor to examine is compliance and the capacity to correctly measure the amount of carbs consumed by children and adolescents with T1DM. Some studies show participants can estimate carbohydrates to within 10-15 g or 15-20% of the actual amount, while others show only half of the participants can accurately estimate carbohydrate amounts [4]. There are multiple formulas or methods for calculating insulin dose requirements using ICR and ISF that are dependent on the parameters of CC [1].

In T1DM, basal bolus treatment is the most used insulin dose method. Basal insulin accounts for about 30 to 60% of daily total insulin requirements. Meals are one of the major factors contributing to a rapid rise in BG. The patient must predict the CC of meals. Nonetheless, the association between carbohydrate intake and the necessary bolus insulin requirement represents a complex reality. Positive outcomes with ACC rely significantly on a patient's adherence to the prescribed regimen, their capability to accurately predict the carbohydrate content of meals, and appropriate adjustment of treatment parameters. The 2014 Consensus Guidelines of the International Society for Pediatric and Adolescent Diabetes (ISPAD) emphasized the importance of nutritional management for children with diabetes [2].

As insulin therapy for people with T1DM is usually modified based on their meals, physical activity, or current BG levels, CC can be done manually or automatically, depending on experience. Using automated bolus calculators (ABCs) instead of manual bolus calculators has been shown to lower HbA1c and improve patient outcomes [30].

CC and insulin calculation in T1DM

The Diabetes Control and Complications Trial (DCCT) considers CC as one of four meal-planning strategies and has proven to be beneficial in assisting participants in achieving glycemic control while allowing them to be flexible with their food choices [16].

Gökşen et al. conducted research to assess whether CC could raise daily insulin requirements in children and adolescents. They found that daily insulin requirements were not affected by CC. Interestingly, the higher insulin need in this scenario was linked to adolescent growth and development, as well as the onset of puberty, rather than CC. The inclusion of fat in carbohydrate-based diets has been demonstrated to increase postprandial insulin requirements and produce postprandial hyperglycemia. T1DM patients should be given an additional dose of insulin three hours after consumption of high carbohydrate and high-fat food because it can lower the concentration of triglycerides and tumor necrosis factor-alpha (TNF alpha), which has been linked to insulin resistance, obesity, and diabetes [1].

ACC is by far the most prevalent method for determining bolus insulin requirements in T1DM. In ACC, the amount of bolus insulin is proportionate to the predicted carbohydrate content of the meal. The DCCT (Research Group, 1993) demonstrated that intensive insulin therapy can markedly reduce the risk of T1DM patients developing long-term consequences associated with chronically elevated BG levels. The DCCT discovered that adequate glycemic control, as measured by an HbA1c level of less than 7.0%, is necessary for reducing diabetes-related microvascular and macrovascular consequences. Even though aggressive insulin treatment is now the gold standard for treating T1DM, only a small percentage of patients reach satisfactory BG levels [2]. Diet adherence is one of the most difficult components of T1DM treatment. On the other hand, nutrition treatment is critical in the management of diabetes, and meal-planning strategies for T1DM place a strong emphasis on the relationship between meal insulin dosage selection and the expected amount of carbs consumed [31].

The most difficult aspect of insulin therapy for T1DM patients is determining the appropriate dose. Insulin requirements are reported to vary widely from patient to patient, as well as from one day to the next and even during the same day for the same patient. As a result, patients must change their insulin doses regularly based on influencing factors such as meal intake, physical activity, and time during the day, although it is challenging and time-consuming to do so. The difficulty in accurately administering insulin is linked to the risk of overdose. Hypoglycemia is caused by injecting too much insulin, which can be life-threatening. Patients must be well trained to perform this activity, with limited support from their primary care practitioner [2,4,32,33].

CC and insulin calculations in T2DM

Most T2DM patients can achieve normoglycemia by using oral antidiabetic medications, while others may require a combination of multiple antidiabetic agents with varying mechanisms of action, along with insulin therapy. As the disease progresses and the pancreatic insulin release response declines, basal-bolus insulinization becomes the preferred treatment for patients with T2DM to achieve adequate BG control. This approach involves the utilization of intermediate-acting or long-acting insulin in combination with regular or preprandial ultrarapid-acting insulin.

The gold standard for T1DM is an insulin dose based on CC. Although many patients find this therapy demanding to utilize on a regular basis, CC can make meal planning more flexible and enjoyable. Some calculations including BG before meals, BG target, ICR, and total CC are necessary to determine the appropriate pre-prandial insulin dose. Due to the complex nature of the medication, as well as the patient's age and education level, such estimates may be particularly problematic for T2DM patients [34].

According to the ADA, even minimal weight loss can help improve glycemic control and minimize the requirement for glucose-lowering medication in patients with T2DM. Diabetic patients benefit from lower BG variability, fewer hyperglycemic episodes, and hence lower HbA1c levels. The total amount of carbohydrates consumed in a meal plays a crucial role in determining the postprandial glucose response. For individuals with T2DM, personalized counseling on self-monitoring carbohydrate intake is essential to enhance meal timing and make informed food choices, as emphasized by both European and American clinical guidelines. Recent studies by Bishop et al. have discovered that patients with diabetes usually misestimate their carbohydrate intake, which has been linked to a higher HbA1c [18].

After a two-part, three-hour training session in ACC, HbA1c dropped by 0.8% (9 mmol/mol) after 24 weeks, with no increase in hypoglycemia or weight gain. They concluded that ACC and insulin bolus calculation are effective, low-cost methods for lowering HbA1c and glycemic variability in people with T2DM who have poor glycemic control despite basal-bolus insulin treatment [35].

Patients' thoughts on the CC method revealed that 66% thought it was challenging, while 34% thought it was useful, allowing them to make more choices and have more flexibility in their meals [35]. It should be noted that although ACC proved to be effective in T1DM patients, the situation is not the same in T2DM patients. According to an RCT, T2DM patients on insulin therapy do not benefit as much from ACC as T1DM patients [18]. Another study showed that T2DM patients who manage their condition through nutrition and exercise, and whether they are using oral diabetic medications, use basic CC as part of their daily diet to help monitor their glucose levels [22]. As the research addressing CC in T2DM is limited, the overall effects are not yet known.

## Conclusions

No final cure for diabetes has been established yet; however, insulin and other BG-lowering agents are used to stabilize blood levels and avoid diabetic complications. The key determinant of postprandial BG in diabetic patients is dietary regulation, particularly carbohydrate intake tracking. CC is a diet management strategy that allows patients to be more flexible in their food choices while also assisting them in identifying BG patterns. CC was not a dietary restriction, but rather an approach that tailors the use of various insulin doses dependent on carbohydrate intake. While CC in T1DM patients showed remarkable benefits, the effects in T2DM are variable and not conclusive. CC has become a hallmark in the management and education of DM patients, with promising results reported in KSA. CC is currently supported as a recommended nutritional approach, in combination with continuous BG monitoring or self-monitoring of BG. New advances in CC may further improve the glycemic control and quality of life for diabetic patients.
